# Food-Dependent Exercise-Induced Anaphylaxis on a Nontraditional Timeline

**DOI:** 10.7759/cureus.86854

**Published:** 2025-06-27

**Authors:** Ruchi R Patel, Ruchi Biswas, Jasmit Walia-Kals, Benjamin Hsieh, Mark Weinstein

**Affiliations:** 1 Internal Medicine, New Jersey Medical School, Rutgers University, Newark, USA; 2 Allergy and Immunology, New Jersey Medical School, Rutgers University, Newark, USA

**Keywords:** allergy, anaphylaxis, case, food allergy, pediatric

## Abstract

Food-dependent exercise-induced anaphylaxis (FDEIA) is a rare but potentially severe allergic reaction that can occur upon eating a specific food followed by exercise. It is triggered by the combination of a food allergen and exercise as a cofactor. We present one of the few documented FDEIA instances with a reversed sequence of ingestion after exercise. A 14-year-old male with no history of food allergy developed anaphylaxis after ingestion of a new protein drink, shortly after strenuous activity. His symptoms included facial angioedema, pruritus, and cough. He was taken to the emergency department and treated with epinephrine, diphenhydramine, and prednisone, with rapid improvement. At follow-up, serum tryptase, C4 and C3 complement, chronic urticaria index, and C074-IgE gelatin values were normal. Skin prick testing (SPT) demonstrated sensitization to trees, grasses, and weeds. Peas, bananas, and strawberries were potentially cross-reactive ingredients in the drink, and negative on SPT. Additionally, the patient had consumed these foods in the past without experiencing a similar reaction. The patient was informed of possible FDEIA and was prescribed an epinephrine auto-injector (EpiPen). He was counseled on avoiding strenuous exercise two hours before and after ingestion of foods that have cross-reactivity to environmental aeroallergens seen on his SPT. In most FDEIA cases, symptoms typically develop after physical activity when the patient has ingested a food to which they are sensitized. Given the possibility of presentation in a reversed sequence, providers should continue to consider FDEIA as a diagnosis when the ingestion event occurs after the exercise event.

## Introduction

Food-dependent exercise-induced anaphylaxis (FDEIA) is a rare yet potentially life-threatening allergic reaction triggered by a specific food allergen or a cross-reactive allergen when followed by exercise [1,2]. FDEIA is an immunoglobulin E (IgE)-mediated food allergy in which consuming a specific food allergen leads to the formation of IgE antibodies that bind to mast cells and basophils. The first case of FDEIA was reported in 1979 [3]. Though the exact mechanism has not yet been elucidated, per a longstanding hypothesis described in literature, exercise acts as a cofactor that lowers the threshold for mast cell degranulation. The subsequent release of histamine and other downstream mediators leads to symptoms of anaphylaxis through a type I hypersensitivity reaction-a rapid, IgE-mediated immune response that occurs when allergens cross-link IgE antibodies bound to mast cells and basophils, triggering their degranulation and the release of inflammatory substances. FDEIA is characterized by an immediate type I hypersensitivity reaction that occurs only when a trigger food is consumed in close temporal proximity to exercise. Notably, the trigger food is well tolerated without exercise, and exercise alone does not cause symptoms in the absence of the trigger food. Common trigger foods include wheat and shellfish. Other cofactors of anaphylaxis include alcohol, nonsteroidal anti-inflammatory drug (NSAID) use, infection, and dehydration [1,4]. Additionally, exercise may alter serum pH, further promoting mast cell degranulation, and over time, increase gut membrane permeability by disrupting epithelial tight junctions [4]. 

Typically, FDEIA presents in individuals who consume a sensitizing food before exercising, within one to four hours of ingestion. However, reversed-sequence cases, where ingestion follows exercise, are exceedingly rare, with only a handful reported in the literature. In a comprehensive review of food-dependent exercise-induced allergic reactions, the reversed sequence represented 1 out of 722, or 0.14%, of cases [5].

Patients at greatest risk are adolescents and adults with a history of atopic disease. Symptom severity and levels of physical exertion vary across presentations. However, the wide range of potential cross-reactive allergens and shared features with other allergic conditions complicate diagnosis [4,6]. This highlights the importance of detailed, temporal history-taking and the difficulty in accurately identifying triggers. Skin prick testing (SPT), specific IgE (sIgE) assays, tryptase levels, and supervised food-exercise challenge testing are key to achieving a diagnosis [2,4]. Still, due to modified threshold reactivity, these tests may not identify the implicated allergen.

Diagnosing FDEIA relies on a clinician’s index of suspicion, which may be further challenged by atypical presentations. Thus, there is a considerable risk of symptom recurrence due to a lack of recognition [1]. We present a unique case of FDEIA in a 14-year-old male, whose reaction was triggered by ingestion of a protein drink following exercise. This reversed sequence presentation exemplifies the need for clinicians to consider FDEIA even when food ingestion follows exercise.

This article was previously presented as a meeting abstract at the 2024 American College of Allergy, Asthma & Immunology (ACAAI) Annual Scientific Meeting on October 25, 2024.

## Case presentation

A 14-year-old male with a history of allergic rhinitis experienced anaphylaxis shortly after consuming a new protein drink following exercise. He had a history of allergic rhinitis symptoms in the springtime and no history of food allergies, anaphylaxis, asthma, or dermatitis. The patient had completed a two-hour session of strenuous basketball practice before ingestion, and within minutes, he began experiencing symptoms that included facial angioedema, eye angioedema, and cough. He was promptly taken to the emergency department, where he received treatment with epinephrine, diphenhydramine, and prednisone. His symptoms improved with intervention. 

At his outpatient clinic visit, laboratory tests, including serum tryptase levels, C4 and C3 complement levels, the chronic urticaria index, and specific IgE to gelatin (C074-IgE), were all within normal ranges. Specific IgE testing to individual drink ingredients was not performed (Table 1). SPT revealed sensitization to environmental allergens, including trees, grasses, and weeds. Specifically, there was positivity to birch, grass mix, and ragweed mix. Other positive environmental aeroallergens include: Ash, white; Beech; Elm, American; Hickory, shagbark; Maple, sugar/hard; Oak mix, eastern; Poplar, white; Sycamore, east.; Walnut, black; Mugwort, common; Pigweed; Plantain, English. The composition of the protein drink included peas, bananas, and strawberries, which have the potential for cross-reactivity with these environmental allergens. However, these ingredients tested negative on SPT, and the patient had previously consumed these foods without adverse reactions.

**Table 1:** Patient's follow-up lab results. Table credits: Ruchi Patel.

Test	Result	Reference
Tryptase	3.9 µg/L	1-15 µg/L
C074-IgE gelatin	Class 0 negative	<0.10 kU/L
CU index	unremarkable	-
Complement C4, serum	17 mg/dL	10-35 mg/dL
Complement C3, serum	115 mg/dL	82-167 mg/dL

Upon discussing the diagnosis with the patient and his family, he was informed of the possibility of FDEIA and was prescribed an epinephrine auto-injector (EpiPen) for emergency use. Additionally, he was advised to avoid strenuous exercise for at least two hours before and after the ingestion of foods potentially cross-reactive with aeroallergens to which he showed sensitization on SPT.

## Discussion

Although food-exercise challenge testing was not performed due to safety concerns, the clinical scenario supports a likely diagnosis of reversed-sequence of FDEIA. The presence of additional cofactors in the anaphylaxis event, such as concurrent NSAID use, dehydration, and temperature, was unknown. Although SPT to the drink ingredients was negative, the possibility of cross-reactivity with environmental allergens remains, given the patient’s sensitization profile. SPT had shown sensitization to tree, grass, and ragweed pollen. There was known cross-reactivity to ingredients in the patient’s protein drink, namely, between tree and strawberry, grass and peas, and ragweed and banana. 

There are a few documented cases of FDEIA in the reversed sequence. Kidd et al. first reported a case in 1983 involving a 39-year-old female who experienced multiple episodes of cutaneous itching, abdominal cramping, dizziness, and weakness following the ingestion of celery, but only when it was preceded by vigorous exercise within two hours [7]. Symptoms lasted one to three hours following each episode. Further evaluation revealed an IgE concentration of 33 IU/mL, with normal complement levels, including C3 and C4. The patient had a history of allergic rhinitis and asthma, and skin testing was positive for ragweed, grass, and tree pollens. 

A comprehensive review by Kulthanan et al. identified 722 cases of FDEIA reactions, with only one reversed-sequence case reported (0.14%) [8]. In this case, Wolańczyk-Medrala et al. described a 27-year-old female who experienced typical and atypical episodes of FDEIA [9]. In these atypical episodes, her clinical symptoms appeared after prolonged moderate-to-high-intensity exercise (lasting 2-5 hours, both indoors and outdoors), followed by consumption of the sensitizing food. The onset of symptoms varied, occurring on average 13 minutes after food ingestion and 17 minutes after exercise cessation. Symptoms included generalized urticaria, angioedema, and syncope. 

Among these rare cases, there is great variation in presentations, complicating clinical recognition. Most cases involved patients with a history of atopic disease and long-term participation in exercise or sports, suggesting that sustained physical activity may contribute to the gut permeability changes observed in FDEIA. Though SPTs were consistently performed to identify the allergen, IgE, tryptase, and inflammatory markers were not assessed in all cases, and when measured, their levels varied across reports. 

Our presented case joins a small group of reversed-sequence FDEIA reports. Each case had varying symptoms and exercise thresholds. The rare mentions of FDEIA cases in the reversed sequence in literature, as well as the varying features of each case, underscore the importance of increased clinical awareness (Table 2).

**Table 2:** Food allergens and cross-reactive species. Table credits: Ruchi Biswas.

Allergen	Cross-reactive species			
	Fruits	Vegetables	Nuts	Other
Ragweed pollen	Banana, cantaloupe, melon, honeydew, watermelon, lychee	Zucchini, cucumber	-	Sunflower seed
Tree pollen [10-12] (Birch)	Strawberry, apple, pear, cherry, peach, plum, apricot, kiwifruit, mango, nectarine	Celery, carrot, potato, bean sprout	Hazelnut, almond, peanut	Soybean, sunflower seed
Grass pollen [13,14] (Timothy, Orchard, Bermuda)	Cantaloupe, honeydew, melon, watermelon, peach, tomato, kiwifruit, and orange	Pea, potato, Swiss chard	Peanut	-

## Conclusions

Such presentations indicate that exercise-induced alterations in immune response and allergen absorption may persist after physical activity has ceased, extending the risk period for FDEIA. This case emphasizes the importance of considering FDEIA in patients presenting with anaphylaxis following post-exercise food intake, especially when the food contains ingredients with potential cross-reactivity to known environmental allergens. Providers should recognize that post-exercise immune and physiological changes may prolong the susceptibility to anaphylaxis upon subsequent food ingestion, especially if the food contains cross-reactive allergens. Thus, providers should continue to consider FDEIA as a diagnosis when the ingestion event occurs after the exercise event. 
